# “*Abusers are Using COVID to Enhance Abuse*”: Domestic Abuse Helpline Workers’ Perspectives on the Impact of COVID-19 Restrictions on those Living with Domestic Abuse

**DOI:** 10.1177/02654075221147203

**Published:** 2022-12-21

**Authors:** Zara P. Brodie, Roxanne D. Hawkins, Chloe MacLean, Jack McKinlay

**Affiliations:** 1 School of Health in Social Science, 3124 University of Edinburgh, UK; 2 School of Education and Social Sciences, 6413 University of the West of Scotland Paisley Campus, Paisley, UK

**Keywords:** COVID-19, domestic abuse, gender-based violence, lockdown, support

## Abstract

**Background:** Mobility restrictions enforced by the UK Government in March 2020 as a response to COVID-19 resulted in those vulnerable to domestic abuse being confined in isolation with their abusers, deprived of safe spaces and many of their usual sources of support. Domestic abuse helplines therefore became an increasingly vital avenue for victim support, seeing a substantial increase in service demand during lockdown periods. **Purpose:** This project examined the nature and frequency of calls received by domestic abuse helplines since the first COVID-19 lockdown period. **Design and Sample:** Through semi-structured interviews with 11 domestic abuse helpline workers across UK services dedicated to a diverse range of populations. Results: Key themes identified through thematic analysis were: (1) Abusers weaponising government guidelines to justify and intensify abuse, and restrictions acting as both a barrier and facilitator to leaving an abusive relationship; (2) A loss of previously accessed support, with users uncertain about what help was available and issues around engaging with new forms of support; and (3) Isolation from social support networks, with callers reporting a loss of respite, lack of emotional and practical support, removal of third-party abuse monitoring opportunities, and subsequent mental health implications. **Conclusions:** These findings will act as a crucial guide for policy decision-making regarding support needs emerging from the pandemic and beyond, highlighting the importance of multi-agency partnerships and clear referral pathways to share the increasing financial burden of domestic abuse amongst services. The longer-term integration of more diverse options for remote support to reduce the risk of detection will be paramount as we emerge from the pandemic, but these should serve to offer a wider range of support routes for abuse victims rather than a replacement for face-to-face provision.

## Introduction

Domestic abuse (DA) refers to psychological, emotional, sexual, physical, and financial abuse within the home, and is a world-wide public health problem (Ertan et al., 2020). The World Health Organisation (WHO) reported that 1 in 3 women worldwide will experience physical or sexual intimate partner abuse in their lifetime (WHO, 2021). Furthermore, data from the annual crime survey for England and Wales indicated that, in the year ending March 2021, around a quarter of domestic abuse-related crimes recorded by police were committed against men (Elkin, 2021), and the Center for Disease Control (CDC) reported that 1 in 3 men will experience sexual violence, physical violence and/or stalking by an intimate partner in their lifetime (Smith et al., 2018).

Mobility restrictions enforced by the UK Government in March 2020 as a response to the coronavirus (COVID-19) pandemic resulted in those vulnerable to DA being confined in isolation with their abusers, deprived of safe spaces or opportunities to physically contact anyone for help or support (Ivandic et al., 2021). Initial reports illustrate that lockdown measures have been associated with a substantial increase in the prevalence and severity of DA (Home Affairs Select Committee, 2020). In the context of lockdown, virtual support provided by DA helplines (DAH) (including call, text, webchat and email services) became an ever more crucial avenue for victim support, in the absence of access to previously available face-to-face or community-based support services. This led to a substantial increase in the level of service provision required from DAH during this time. For example, the UK charity Refuge reported a 700% increase in traffic to their support website and a 120% in calls received to their helpline in April 2020, compared to April 2019 (Refuge, 2020a, 2020b).

Stay-at-home orders implemented by the UK government unintentionally elevated the threat of abuse for those living with a violent or abusive partner. Government restrictions led to isolation from social networks, and prevention of normal work-related or social activities, both of which are commonly seen to be key elements of a DA scenario (Piquero et al., 2021). This potentially provided a unique opportunity for abusers to use governmental guidance to add weight to their controlling and coercive behaviours. As such, the crime survey of England and Wales reported that, between March and June 2020, there was a 7% increase in police reports of DA, with 21% of all offences recorded by police being DA-related; an increase of 5% from the previous year (Office for National Statistics, 2020b). Given that many elements of domestic abuse overlap with other offences (e.g., violence against a person), the police are required to indicate when a recorded offence is seen to be DA-related. In this same period between March and June 2020, there was seen to be a 9% increase from 2019 in violence against the person offences that were indicated to be DA-related, highlighting that the risk of physical harm was especially elevated for those living with an abusive partner during the initial stages of lockdown (Office for National Statistics, 2020a). In the same report, it was also found that figures for DA-related incidents that fell within all other offence groups also increased, apart from sexual offences, which decreased by 3%. There was also seen to be a 12% increase in victim support referral for DA cases, and a 65% increase in calls, compared to pre-pandemic, to the National Domestic Abuse Helpline. Between April and June 2020, the Metropolitan Police reported an 11.4% decrease in reported abuse perpetrated by former partners (Ivandic et al., 2021), suggesting that in some cases restrictions may have been a protective factor. However, an 8.1% increase in current partner abuse was also found, which highlights that those living with an abusive partner were put at greater risk.

While men who are victims of domestic abuse are considerably less likely to report to police for several reasons (e.g., fear of not being believed, shame and embarrassment due to gender norms faced by men; Walker et al., 2020), research examining engagement with the targeted domestic abuse charity Respect Men’s Advice Line during the pandemic indicates that calls from men regarding abuse from current and former partners (the majority of which were women) increased during lockdown, with the service receiving 3527 calls between April and June 2020 (the highest since the service launched in 2007). It was found that callers primarily reported physical, verbal, and financial abuse, and an increase in coercive and controlling behaviours enacted through the lens of government guidelines – such as forbidding the victim from ever leaving the home, even for reasons deemed acceptable by the government (e.g., grocery shopping).

There are several reasons as to why the COVID-19 lockdown measures enforced created an environment conducive to DA. Firstly, for the large part, victims were forced to stay at home with their abusive partners. This consequently provided abusers with increased opportunities to aggress and offered victims fewer avenues to retreat from the abusive environment (Women’s Aid, 2020b). Families, regardless of the presence of abuse, also faced several pandemic-related stressors, with many reporting ‘pandemic burnout’ due to the exacerbation of prior stressors and the addition of new worries related specifically to the virus (Marsh, 2021). This includes concerns regarding threat and risk of contraction, job security and financial stability, and the ability to access appropriate healthcare and childcare, all of which had implications for psychological health and wellbeing (Fiorillo & Gorwood, 2020; Jia et al., 2020). These additional stressors put undue strain on relationships generally, with research indicating that couples reported higher levels of partner interference, negative emotions towards interacting with their partners and an increase in relationship turbulence during the outbreak, compared to pre-pandemic (Goodboy et al., 2021). Relationship satisfaction, intimacy and appreciation were also seen to decrease, while relationship conflict increased, from pre-to mid-pandemic, which was found to contribute further to the level of stress experienced by both partners during lockdown (Pauley et al., 2022). For those living with DA, these cumulative stressors served to exacerbate already existing tension and strain within the home, creating what is being referred to as a ‘perfect storm’ (Women’s Aid, 2020a). Indeed, factors such as the inability to maintain social connections and increase in financial and economic stress have been associated with an increase in DA rates (e.g., Arenas-Arroyo et al., 2020; Béland et al., 2020).

Pre-pandemic research suggested that DA victims were more likely to seek help and support through informal means (e.g., friends and family) and when positive, these informal routes could increase the likelihood of formal reporting of abuse such as through police or victim support services (Goodman et al., 2005; Plazaola-Castano et al., 2008). However, during lockdown, the opportunities to informally disclose abuse to social networks, or for those close to the victim to detect abuse, were greatly minimised due to enforced social distancing restrictions. This meant that those wishing to disclose DA had to engage with more formal support services in the first instance. However, government restrictions saw the scaling back, or complete withdrawal, of many face-to-face services, including community-based support services for those seeking refuge (Women’s Aid, 2020a). In line with this, while police reports of DA declined during the pandemic, remote engagement with victim support services (e.g., web traffic, helpline calls, text, and email contact) substantially increased. Exploring the nature of contact received by DAH is therefore a salient avenue for developing a better understanding of the experiences of those living with abuse during the COVID-19 pandemic.

### The Current Study

The increase in prevalence and severity of abuse experienced during the pandemic will have long-lasting negative impacts for DA victims and for DAH workers providing support. Reports suggest that UK support for DA victims was inadequate prior to COVID-19 (Bradley, 2020), indicating that while this problem may have been exacerbated by lockdown, it is not localised to the pandemic and will continue to require urgent attention post-COVID. A report released by the Home Office in 2019 (Oliver et al., 2019) estimated the annual social and economic cost of DA in England and Wales (year ending March 2017) to be approximately £66 billion. The lingering impact of the surge in DA cases and severity during COVID-19 lockdown will most likely see an increase in these costs as DA victims and the organisations that support them struggle to recover from this unprecedented situation. Through semi-structured interviews with DAH staff, this study will provide an important understanding as to how the nature of abuse changed during lockdown and the role played by DA helplines. This will be vital in the development and refinement of long-term strategies in the UK for improving support provision for victims and ensuring helpline staff have the tools required to provide adequate support.

## Methods

This study was conducted in April 2021 as part of a broader three-phase UK-wide study examining the impact of COVID-19 restrictions on those living with DA and the experiences of helpline staff supporting them during this time. This paper focuses specifically on the findings from the first phase of the study that examined the nature of calls received by DA helplines during COVID-19 lockdown periods through a series of virtual semi-structured interviews with DAH staff.

### Participants

Participants were recruited purposively through five UK-wide DA support organisations, all of which are registered charities providing free and confidential support to victims of DA. The only inclusion criteria were that participants must be over the age of 18 and have worked in a paid capacity on the helpline during the pandemic. All organisations that took part were referral/listening services, who primarily offer guidance and advice, and signpost service users to appropriate services for further help (e.g., refuge, counselling support, financial aid). Prior to the pandemic, all the charities offered face-to-face drop-ins (either on-site, or in other safe places in the community – e.g., pharmacy consultation rooms) as an alternative to reaching them via call or email. During the lockdown restrictions, all support was shifted to virtual platforms.

The final sample (see Table 1) consisted of 11 participants (10 identified as cisgender women, one identified as a cisgender man; aged 24–56, M = 39.6, Mdn = 39) from helplines targeting women, men, and LGBTQ + abuse victims. All participants who agreed to take part initially followed through with their participation. This number was deemed appropriate based on recommendations to reach saturation (Guest et al., 2006) and is comparable to other recent publications that examine the experiences of charity helpline staff (e.g., Taylor et al., 2019,*N* = 10). The majority of these services offered both in-person and virtual support pre-pandemic, but during the pandemic all support became remote (with helpline staff working from their own homes), allowing service users to reach out to these organisations using helpline numbers, webchats and emails. The UK Government launched a national awareness campaign (#YouAreNotAlone) to increase the visibility of remote support services for DA victims amidst the pandemic, signposting to helpline numbers, email contacts, and websites for more information.

**Table 1.** Participant Characteristics (*N* = 11).

Pseudonym	Age	Gender	Victim demographic	Months in post	Contract
Alfie	27	Man	Men	7	N/D
Anna	32	Woman	All victims	24	Full-time
Bianca	N/D	Woman	All victims	15	Full-time
Chiara	29	Woman	Women	96	Full-time
Debbie	46	Woman	Men	36	Full-time
Emilia	38	Woman	All victims	18	Part-time
Freya	55	Woman	Women	54	Full-time
Georgia	40	Woman	Men	24	Part-time
Harriet	49	Woman	Women	17	Part-time
Isabelle	56	Woman	Women + LGBTQ+	66	Full-time
Jessica	24	Woman	Women	6	Full-time

*Note*. N/D = Not Disclosed.

### Data Collection

Following ethical approval from the University [blinded] review board, several UK-based DA helplines used their internal staff emailing system to circulate an online survey link which led them to an information sheet, consent form and availability request should they be interested in taking part. Those staff were then contacted by email to confirm their interview timeslot and provide links to access a virtual password-protected meeting room. All interviews were conducted by the project Research Assistant (RA), who at the time held a BSc in Psychology (Hons), had completed a Carnegie Vacation Scholarship and was in the process of completing an MSc in Research Methods in Psychology. The RA had extensive experience conducting interviews through their own research, and in previous RA roles with the research team. The RA was unknown to the participants prior to commencement of the study. Before the interviews took place, participants were only informed that the RA was a man, and that they could request a woman interviewer should they prefer. However, no participants requested this.

All interviews were conducted using Zoom video call software, and all participants agreed to have their interview video-recorded for transcription purposes. Only the interviewer and interviewee were present in the virtual meeting room. Following demographic questions (age, gender, location and target demographic of charity, time in post and contract type), interviewees were asked to reflect on the following questions: (1) Can you describe the nature of the calls you have received during lockdown/pandemic? (2) Do you think the nature of the calls has changed since covid-19 restrictions? If so, in what ways? (3) Can you tell us about any calls that mentioned the impact of being isolated from others such as friends, family, or other social support networks? (4) Can you tell us about any calls that mentioned the impact of potential lack of access to previously available community-based support services? (5) Did any calls relate to the nature and impact of other stressors related to the pandemic (e.g. financial, health-related)? Note that *n* = 2 participants (Alfie and Jessica) did not have pre-pandemic helpline experience, and so were not asked to provide insight to Question 2.

Interviews lasted between 60 and 90 minutes, and any instances where any identifying information was provided by a participant, such as the name of their organisation, this information was removed before transcript files were finalised and shared amongst the research team. Following participation, participants were emailed a £10 Amazon voucher to thank them for their time.

### Data Analysis

Interview data were analysed using an inductive and data-driven approach to thematic analysis (Braun & Clarke, 2006) that involves several steps. First, all authors independently read and familiarised themselves with the transcripts (139 pages, reflecting 562.63 hours of interviews in total) and produced initial codes and potential themes. A collaborative approach was then undertaken whereby the team discussed the initial 18 codes, identified overlaps or similarities, and agreed final codes that were then applied to the transcripts independently to determine dominant themes. This investigator triangulation approach is recommended to reduce analytical bias and enhance credibility of the results (Wilson, 2014). As advised by Braun and Clarke (2006), we then came together to create a thematic map, allowing for the visual identification of concept cross-over between analysts, and standardisation of terminology, definitions, and structure of the major themes and sub-themes identified. This map was then used by the team to finalise analysis of each transcript and create a data matrix whereby relevant and meaningful quotes to reflect the themes and subthemes identified were stored under theme and sub-theme headings within an excel document shared by the research team (Gale et al., 2013).

## Results

Thematic analysis identified three overarching themes, each with corresponding subthemes (see Table 2).

**Table 2.** Themes, Subthemes, and Sample Contribution.

Themes	Definition	Subthemes	*n*	%
1. The impact of lockdown on DA experiences	Changes in the nature of abuse and victims’ perceived ability to cope	• Lockdown-related changes in abuse	9	82
		• Breaking point	7	64
2. Pandemic-related changes to service provision	Change in support provision, and benefits and challenges in accessing this support	• Issues accessing support	10	91
		• Adjusting to entirely virtual support provision	8	73
3. Isolation from social networks and support	The impact of locked-down related social isolation on victim experiences and wellbeing	• Lack of contact with family and friends	11	100
		• Lack of respite	7	64
		• Mental health implications	10	91

*Note*. n = number of participants who contributed to each subtheme; % = percentage of participants who contributed to each subtheme.

### Theme 1: The Impact of Lockdown on DA Experiences

The first theme focuses on the impact of lockdown measures on the abuse experienced of helpline callers. This is broken down into two subthemes: *Lockdown-related changes* in abuse, and *Breaking point*.

#### Subtheme A: Lockdown-Related Changes in Abuse

Several participants felt that the severity of abuse itself did not necessarily increase as a result of COVID, but rather the frequency and inability to exit the situation worsened: “*Not that it is worse, because abuse is abuse, but just that the frequency and the inability to get away from it, I think, was affecting women more.” (Chiara)*

Alfie agreed with this notion, arguing that they did not feel there was an increase in severity of abuse, but that there were *“just less options for people”*. However, there was agreement across participants that the pandemic did alter the way in which abuse was carried out. In many cases, pandemic-related restrictions put in place by the government were utilised by abusers as a tool to add weight or justification to abusive behaviours that had already been present:

*Their partners are using that as a way of coercive control. And so we're seeing things like, ‘I'm not saying you can't go out because I don't let you, I'm saying you can't go out because you know it's dangerous, it's not safe for you because you're vulnerable’. So partners using coercive control as a way to… kind of make the isolation even more so. (Anna)*

This point was raised by several participants, with Harriet stating that abusive partners were touting the message that *“the government is saying that you have to stay at home with me”*. Perpetrators were therefore believed to be using government guidelines to diffuse responsibility for their controlling behaviours, putting additional pressure on victims to comply. On the other hand, participants also reported that some abusers would purposefully disobey government guidelines as a means of causing distress in victims:

*Women in particular were saying that they were obeying the lockdown measures as much as they could. And, and their overwhelmingly male partners, were disregarding the measures. So, going into other people's houses, going to the pubs when you were still able to do that, and not washing their hands and sort of disregard and disrespecting those measures specifically as part of the emotional abuse. (Anna)*

Isabelle felt it was clear that perpetrators were *“breaking the rules to scare and abuse”,* and even stated that *“some women were washing their kids down after seeing dad”* for fear that they may have come into contact with the virus when in their car, highlighting that some abusers viewed government guidelines as opportunities to inflict further abuse onto their victims.

Again, while participants mostly indicated that the type of abuse experienced remained relatively consistent with that prior to the pandemic, often the context of COVID provided a platform for certain abusive behaviours to flourish and intensify. Debbie referred to abuse as having *“changed to a COVID way”*. This was often related to the increased proximity between the victim and abuser, where abusive and controlling behaviour, such as monitoring behaviours, sharply increased:

*People have been, you know, controlled and monitored in terms of making phone calls or computers for the web chat and email service seem to be more scrutiny over things like that, where people will be monitored, and they were worried about sending emails or sending web communication and that being seen by their partner. (Debbie)*

This illustrates that the increased monitoring opportunities for the abusive partner meant a further reduction in opportunities for the victim to reach out for help or support. Harriet also emphasised that this constant proximity meant that the abuse, or threat of abuse, also became more constant:

*The victim and the perpetrator are just in the same property quite often and in close proximity. So, the psychological, emotional abuse was just kind of constant. I did have some callers saying that the perpetrator might not be doing anything. Like they could wake up and not speak to them all day, or see them because they were in another room working. But the thought of the abuse was there. (Harriet)*

Freya noted an increase in the number of Multi-Agency Risk Assessment Conference (MARAC) referrals dealt with by their organisation, which was centred around women victims and their children during lockdown:

*Our referrals to MARAC have definitely increased. So that's a very good indicator of the level of risk that has risen in the last year. The numbers to MARAC have been quite high. We used to do meetings where you would hear 6–7 cases maybe on average. These days we've been having meetings, it has been tailing off again, but there have been one’s of 14–15 cases, which is really really unheard of*.

MARACs are a multi-agency approach to managing DA cases where the victim has been deemed as at high risk of serious harm or homicide, and an increase in such referrals during lockdown demonstrates the increased risk faced by those living with DA during the pandemic. Isabelle also made reference to MARAC referrals through their organisation, but did not indicate whether these had specifically increased during lockdown.

#### Subtheme B: Breaking Point

For some victims, participants indicated that the increased intensity of abuse during lockdown acted as a “wake up call” that instigated their decision to flee the relationship. This was often due to an increased escalation of abuse and no longer feeling able to manage:

*I think a lot women are considering more extreme changes like refuge, or like presenting as homeless, when they would never have done that before because it was so much more manageable. So, the escalation of things has been a lot quicker than what that before COVID. Before it would be that you would try a lot of smaller things, you know, safety plan and risk assess with them, you know, do small little things that will keep them safer with the woman feeling that she was managing it better. But now with COVID and all those other external COVID-related factors, it has made it a lot harder to manage. (Jessica)*

This changed the nature of support needed by the DAH staff, with help becoming more urgent and critical as callers reported reaching “*breaking point*”:

*It feels like callers and women are wanting out now and need a plan of action instantly. So, it gone from 0 to 100 a lot quicker and they're just saying ‘I need out of the house now’, and that is really rare that you get a woman literally say they need out immediately, usually they just want some information to weight up their options. But a lot of the phone calls now, I get the sense they are at breaking point. It like ‘I need a solution today and right now’, rather than, ‘can you run over some solutions for me and I’ll think about it’. (Jessica)*

This illustrates how, for some victims of DA, being forced to confront the reality of their abusive situation during lockdown acted as a breaking point that led them to make more extreme or urgent exit decisions than they perhaps would have in the past. In line with this, participants reported that more callers executed plans to leave the home than they had experienced in the past:

*One thing from lockdown is that women realised that like things escalated and got worse and actually moved out. We got a lot of referrals of women who were wanting to flee. So yeah- before they may just be living their life and whatever, but now they realised the situation they are in and that it isn’t right or safe for them and their children. So, maybe before they were going out to their jobs and whatever, but now living in that environment 24/7, it really made them think like ‘oh I really need to get out of here’. (Isabelle)*

Lockdown measures therefore appear to have contributed to an increase in urgent at-risk cases dealt with by support organisations, with many believing that abusers weaponised government restrictions to their advantage. However, in some cases, this increased risk of harm acted as a wake-up call to victims, instigating more immediate exit decisions.

### Theme 2: Pandemic-Related Changes to Service Provision

The second theme reflected changes to the support available to those living with DA during the pandemic, divided into two subthemes of *Issues accessing support*, and *Adjusting to exclusively virtual support provision.*

#### Subtheme A: Issues Accessing Support

Participants highlighted the implications of the pandemic and associated restrictions on previously relied on availability and access to support, including mental health and other community-based support services:

“*A lot the mental health services that people may have already been interacting with, they just stopped doing the first lockdown… All face-to-face support stopped and everybody went into crisis.” (Debbie)*

Debbie explained how the withdrawal of these services led those living with DA into a state of crisis. A loss of support was not the only concern raised by callers, with limited access to support for other household members such as children also being problematic. For example, Bianca stated that “*someone was talking about that she and her daughter had been accessing counselling, and that had stopped with the lockdown*”. Participants explained that even in instances where services remained open, changes to the nature of their provision made it more difficult for victims to engage with them:

*I mean we weren't open the way that we would be. At one point we weren't allowing drop-ins, and that's a big thing for women to just be able to pick up their stuff and come into the office where they know they're in a safe place and we go from there. That wasn't even an option so that added to woman’s stress about how to get support and where to turn to. I know a lot of services like ours, it was the same. So, I think, leaving a relationship like that is hard enough, but it's trying to leave a relationship like that when those options aren't there. (Jessica)*

Jessica highlights that those most vulnerable to the removal of in-person support were victims on the cusp of removing themselves from the abusive relationships, as they no longer had a safe space to flee to. Participants were consistent in highlighting how this withdrawal of support left victims unsure of where to turn, and unclear as to what support was actually available during lockdown:

*They were getting messages from the media like you can’t call your doctor because they are too busy, you can't call the police because they are too busy, you can't call the ambulance because they're too busy. So, there was literally not knowing where to go. (Emilia)*

The above quote demonstrates that widespread media coverage of the additional pressure put on support services such as the NHS and the police left victims feeling as though they had nowhere to turn for support. Emilia explained that this was especially prominent in instances where callers had received shielding letters from the government, urging them to self-isolate due to health-related vulnerabilities: *“they didn’t necessarily know how to access the support they required.”*

As highlighted by the participant quotes above, the reduction, change in format, or complete withdrawal of previously accessed support left DA victims with little options to seek formalised help and impacted on their ability to flee the abuse. This reluctance to seek support was also exacerbated by messages from the Government and media emphasising how overrun these services were during the pandemic, which made callers hesitant to add to that problem.

#### Subtheme B: Adjusting to Exclusively Virtual Support Provision

The next subtheme is centred around issues associated with the shift from access to face-to-face support to the exclusive use of virtual platforms, which included telephone, email, and webchat-based support. Participants highlighted both challenges and benefits associated with this transition. Negative elements included both a lack of access to resources required to engage with certain elements of their online support provision, including therapeutic services, with Jessica explaining that some callers simply *“didn’t have access to a laptop”*. However, some organisations were given additional funding by the government to assist those with resources limitations:

*We also got a lot of money so we were able to give women who didn't have a laptop a Chromebook. So, we gave them laptops and stuff from the government. So, women could link in then to do the group work but virtually. (Isabelle)*

However, participants were also concerned as to whether online support could effectively meet the needs of the diverse population that they come into contact with: “*When I think about people that may have learning needs, or disabilities or older groups of people whose first language isn’t English, I’m not sure how we’re meeting the needs of those groups of people.” (Debbie).* Debbie showed concern that online support was inherently limited in its utility for the groups highlighted above, which meant that they were left unsupported during the pandemic. This sentiment was echoed by Georgie, who said that *“not having face-to-face interaction, it’s kind of one step harder for them to seek help for many reasons maybe because of their disability or condition”.*

On the other hand, accessing support online simply *“wasn’t achievable”* for some women, according to Jessica. This was most commonly attributed to the reduced “*window of opportunity*” for victims to reach out in this context, where they are limited to perhaps only a few minutes where their partner is not present: “*We have been getting those types of calls where they are saying ‘I have 5* *minutes or I have 10* *minutes to quietly tell you what’s going on for me and I need a solution now’.”* Jessica indicated that this sense of urgency was in stark contrast to the nature of the support they provided prior to the pandemic, where victims could visit their service face-to-face, and support was “*a bit more planned*”.

Several participants also indicated that being confined to remote support meant that many victims struggled to reach out by phone due to their abusive partners being in such close proximity, but the increased provision of other online support methods, such as email, seemed to help combat this to some extent:

“*We noticed that, you know, more and more people were saying that they weren't able to contact and that speaking on the phone was difficult. And initially, phone call numbers dropped and a massive surge in email communication.” (Debbie)*

Despite the limitations identified with virtual support provision, participants did highlight some benefits of this alternative to face-to-face support, including eliminating physical access issues such as travel:

*For some people in rural areas, it’s very hard for them to get to these services. So that used to be a problem for them anyway. But like I say they were the ones that benefited more from that virtual thing because they didn’t have to travel. (Isabelle)*

The move to online support also meant that victims could navigate their engagement around other commitments, which removed barriers to accessing face-to-face services such as a lack of appropriate childcare during organisational office hours:

*Getting childcare is a big thing for any community support for callers. Maybe the support is only available after school hours, and women can’t get childcare sorted so can’t go. But with the virtual thing, maybe the kids were upstairs or in a quiet room somewhere, and so women didn’t have to get childcare… (Isabelle)*

Isabelle also expressed that the virtual support available was crucial for many victims to alleviate feelings of isolation to some extent: *“Just even feeling that they weren’t alone. I mean if we didn’t have virtual stuff, I don’t know how some women would have coped”.* This suggests that the transition from face-to-face support to virtual platforms brought with it both benefits and difficulties for those living with DA.

### Theme 3: Isolation from Social Networks and Support

The third theme reflected the experience and impact of isolation from social networks and support due to pandemic-related restrictions. This theme is divided into three subthemes: *Lack of contact with family and friends, Lack of respite,* and *Mental health implications.*

#### Subtheme A: Lack of Contact with Family and Friends

Participants indicated that the government guidelines which restricted face-to-face contact between households meant that many victims lost a key source of social support from friends and family members:

*They would have been seeing the mum once a week for help with childcare and that was like a lifeline and that all fell away, or people are saying like just about the mental health. Again, like contact with others or like a sort of… some sort of support network would have been part of the coping within the abusive situation and that that was all gone. (Bianca)*

As highlighted by Bianca, this had practical implications in terms of childcare support but also impacted on victims’ mental health, making it more difficult to cope with their home situation. Chiara also noted that this could at times lead to further reliance on the abusive partner to provide childcare support, which added pressure for the victim and gave the perpetrator more control.

Participants who worked for men victim-focused charities noted that the restrictions on contact with social networks significantly reduced their ability to remove themselves from the abusive situation when abuse became severe:

*I think before I had the sense that people in that kind of situation would have stayed with a family member or, or, you know, had the option at least to go and kind of crash on a friend’s sofa. (Alfie)*

This isolation from friends and family was also reported to impact on men’s opportunities to open up or share their experiences with those close to them: “*The isolation side of it was not being able to see people, not being able to visit and speak to them in confidence.” (Debbie)*

One of the most significant issues raised by participants in relation to reduced contact with friends and family was the absence of any kind of external abuse monitoring. Participants who worked for both women and men-focused organisations highlighted the importance of victim monitoring by close others as a means of identifying risk or need for intervention, as seen in the following two quotes: “*It’s a safety layer removed. So if there is abuse going on somewhere in their lives then it’s hidden just because there’s not anybody else physically around regularly checking out what’s going on.” (Georgie)*

*A woman's dropping off her kids to her mum’s every day for childcare, her mum might pick up on the fact that she's not looking like herself, she’s not presenting very well and ask questions, and she will maybe disclose to her mum, but she's not getting those opportunities now. (Jessica)*

The quotes above emphasise the reduced opportunities for those close to the victim to notice that abuse is occurring and offer support. Bianca expanded on this by explaining how this left victims with a “*lack of reality checking*” or feedback from others around them to verify that their abusers’ behaviour was “*not normal*” and “*not acceptable*”.

*When they are isolated the only voice they're getting is the perpetrators voice about how worthless they are, and like how everything is their fault. And so, yeah, they are just in this kind of echo chamber. So, yeah, they don't feel like they deserve help because they've been told that they are the problem. (Bianca)*

Participants raised concern that this also often left callers uncertain as to whether what they were experiencing actually constituted domestic abuse or not, given the lack of feedback from others. Isabelle further suggested that the removal of these external third-party monitoring opportunities meant that the *“perpetrator thought that they could get away with it more”,* thereby further contributing to an escalation in abuse.

#### Subtheme B: Lack of Respite

Another prominent subtheme was the lack of respite from abuse. Participants highlighted that often those living with abuse felt able to cope with their situation prior to the pandemic because they had breathing space from the abuser at certain times. However, given that both parties were often working from home during the pandemic, these opportunities were removed:

*So previously, a way for them to cope, or get some sort of a respite would be them going to work or the perpetrator going to work. And there being some point in the day where they weren’t crossing paths and so while the abuse experience is always there, they're not kind of actively at risk for that person. (Anna)*

*There has also been a lot of women who have almost accepted that they're going to be in an abusive relationship because they could and were managing it quite well because they had down time from that person. But now they are saying ‘no, I can't do this when it’s 24/7’. (Jessica)*

This was echoed by Debbie (men-focused organisation), who suggested that being confined in the home with the perpetrator, with no opportunities for respite, *“contributed to more conflict and other types of abuse.”* Harriet also argued that the increased time spent in contact with the perpetrator increased the risk for more physical forms of abuse specifically:

*Whereas, I suppose, if he was going out to work and coming back, then the abuse is only when he comes back home. I mean not necessarily because the abuse could carry on when they are at work, you know like psychologically. (Harriet)*

Participants also highlighted how callers were unable to engage in activities that often represented important protective or coping mechanisms in helping them deal with their abusive situation:

*They don’t have their usual protective factors. Like they aren’t able to go out to the park for a walk or go to the gym, you know, there's nothing in place to kind of help them process the things they've been through. So I think their usual coping mechanisms are not available to them anymore. (Jessica)*

#### Subtheme C: Mental Health Implications

Several participants made reference to the impact of isolation on the mental health of callers. Compared to pre-pandemic, many indicated that calls related to mental health increased, and this was true for charities targeted at all genders:

*There was a massive increase in mental health issues, and many people spoke about not feeling that they were able to cope, feeling low mood and depressed. We had a much higher number of people talking about suicidal thoughts and self-harm. And there was a lot more crisis management in terms of managing people's emotional wellbeing. (Debbie)*

Jessica indicated that more referrals were being made to *“mental health services like Samaritans and BreathingSpace, so places that are more for mental health support.”* Chiara also explained that their organisation had to adapt from primarily being a signposting organisation, prior to the pandemic, to engaging in *“far more safeguarding work”* with victims disclosing mental health issues and suicidal thoughts. While the only participant to do so, Jessica did note that the pandemic also potentially had mental health implications for the abusive partner, which further compounded the abuse within the home: “*…on top of that their partner is probably having the same anxieties and stresses about lockdown, just like everyone else.”*

Overall, forced withdrawal from friends and family members brought significant difficulties for victims, both emotionally and practically. This accumulation of factors further exacerbated mental health issues and elevated risk of harm, putting further pressure on support organisations to respond to those in crisis.

## Discussion

This study aimed to examine DAH workers’ perspectives on DA in the context of COVID-19 and the restrictions enforced by governments worldwide as a response to the pandemic. Given that the pandemic has been associated with an increase in DA incidents and/or severity across the globe (Piquero et al., 2021; Ravi et al., 2021), it is imperative that we develop an understanding of how COVID-19 impacted on those living with abuse. The three overarching themes identified help provide insight to ensure that appropriate care and support is established and put in place as we emerge from the pandemic and begin to deal with its long-lasting impact on this vulnerable group.

While there was little indication that the severity of abuse itself increased, there was certainly agreement that the frequency and constancy of abuse was amplified during lockdown. Our findings align with and emphasise recent reports of ‘pandemic-specific abuse’ (Davidge, 2020; Gregory & Williamson, 2021), where perpetrators were perceived to capitalise on the pandemic context in their abusive tactics. DAH staff consistently provided examples of how they heard from callers that abusers were “using” government restrictions to add weight and justification to their coercive and controlling behaviours. Helpline staff believed that this allowed abusers to shift responsibility for their abusive demands and add additional pressure for victims to comply. It is important to note here that, during the pandemic, the UK government put in place a formal exception to the stay-at-home orders for victims living in an abusive household, which meant that – legally speaking – victims of abuse were still able to leave the household to seek support from friends and family, to seek formal support or to flee harm (Health Protection (Coronavirus, Restrictions) (England) Regulations, 2020). Furthermore, households in the UK were also allowed to form ‘childcare bubbles’ (Department of Health and Social Care, 2020). This meant that parents living in a household with children under the age of 14 could form a childcare bubble with friends or family from another household to provide support with informal childcare. Despite these exemptions, there was no mention by any participants regarding their service users acting upon them – on the contrary, almost all participants provided several examples of callers reporting that their abusive partner was preventing them from leaving the home at any time and preventing contact with friends and family. This may in part be down to the lack of any kind of public awareness campaign to highlight the existence of these exceptions, which meant the only way an individual could become aware of their rights as a victim of abuse was through in-depth engagement with the published regulations or through advice received after reporting abuse (e.g., to the police or to support services). This highlights a considerable limitation in the government’s response to DA during COVID-19, which left victims believing they had no choice but to remain isolated at home with their abuser. Some abusers were also reported to be purposefully ignoring COVID guidelines to cause their victims fear and distress around contraction for both them and their children. The restrictions themselves were also said to provide more monitoring opportunities for abusers, which made it even more difficult for victims to reach out for support.

Interestingly, some DAH staff believed that, for some victims, the context of the pandemic acted as a breaking point, where the constant threat or increased frequency of abuse, and heightened risk of exposure for children within the home, led them to either reach out for support for the first time or make the decision to exit the relationship. This is supported by previous research that highlights fear surrounding child safety (Peterson et al., 2005) and perceived increase in risk of serious harm to oneself (Costanza Baldry & Cinquegrana, 2021) as prime motivators for seeking help and accessing formal support services. This suggests that, while the motivators for leaving an abusive relationship did not change during the pandemic, the sudden spike in abuse exposure resulting from stay-at-home orders may have served to fast-track decisions to leave in some cases.

Issues were also identified surrounding changes made to support provision during the pandemic. The government restrictions put in place to combat the spread of COVID-19 meant that the majority of support organisations were forced to close their doors to the public, removing all face-to-face engagement opportunities. While most began to offer various remote alternatives, including increased helpline opening hours and a stronger focus on the use of email, webchat, text and videocall support, DAH staff proposed that using such platforms as an exclusive mode of support delivery brought about new challenges for those seeking support. In many cases, staff thought that victims were left confused and unclear on what support remained available to them, especially in terms of in-person refuge options, and this was heavily reinforced by media messages relaying the additional pressures placed on support services during the pandemic. Victims therefore felt trapped in their abusive situation (Oppenheim, 2021).

Domestic abuse helplines staff also felt concerned that the transition to purely online support meant that some more vulnerable groups were left unsupported, due to either a lack of resources required to engage (e.g., laptops), additional support needs that made engaging with technology more difficult, or because contact proximity to their abusive partner meant there was little space or time for them to engage without detection. It is perhaps unsurprising that the pre-existing issues around support seeking for those experiencing technology-facilitated abuse (e.g., monitoring of devices; Douglas et al., 2019) were heavily exacerbated by lockdown restrictions (Pfitzner et al., 2020).

However, certain advantages were acknowledged regarding accessing support online versus in-person. For example, staff saw virtual support as removing barriers to engagement for those who were restricted by childcare commitments or travel. Indeed, prior research has emphasised geographical isolation and transportation limitations as a prominent barrier to accessing traditional support for victims in more rural areas (Youngson et al., 2021) and as such, the increased provision of remote services may have in fact proved beneficial in such cases. For those who did engage with the remote services on offer during lockdown, callers expressed that online engagement offered some relief from feelings of isolation and loneliness during lockdown and afforded them more opportunities to seek support without detection while their abuser was in the home (which made speaking on the phone more difficult). However, as support organisations begin to evaluate the utility of integrating increased remote support provision on a more permanent basis, it is crucial that the barriers this mode of engagement presents for those living with technology-facilitated abuse and those without appropriate technological resources are at the forefront of such discussions.

Domestic abuse helplines staff reflected on the enduring impact that isolation from social networks and support had on their callers. For many victims of DA, interactions with friends and family members is a fundamental source of support that helps them cope with their abusive situation, either by providing emotional comfort or more practical support including childcare or a safe space to which they can retreat (Klein, 2012). For some, this lack of previous support from social networks was seen to increase reliance on their abusive partner for support, relinquishing further control to the perpetrator, highlighting the important role played by informal third-party support for those living with DA. One of the most prominent consequences of reduced third-party contact was the absence of external abuse monitoring. Those close to victims no longer had opportunities to detect instances where abuse was happening, had worsened, or to provide input to the victim regarding the inappropriateness of their abuser’s behaviour. One participant referred to victims’ being stuck in an “echo chamber”, where the only voice being heard was that of the abuser telling them it was their fault and that the abuse was deserved. This lack of reality checking left some victims unsure as to whether what they were experiencing was indeed abuse, or whether it was normal. Family members of those living with DA have similarly reported that COVID-19 lockdown impeded their ability to assess the level of danger faced given that there were no opportunities to identify cues that the abuse was still ongoing or had worsened (Gregory & Williamson, 2021). Informal support seeking from friends and family often marks an important first step in pursuing help for DA victims, whereby positive informal support seeking experiences increase the likelihood of progressing to engage with formal support services (Goodman et al., 2005; Plazaola-Castano et al., 2008). The removal of this option during lockdown may have consequently delayed the process of help-seeking or escape from an abusive relationship for some.

Call-takers also highlighted how a lack of respite during COVID restrictions, including hobbies or normal working patterns for both the victim and abuser, contributed to the escalation in conflict within the home due to chronic proximity (Office for National Statistics, 2020). Many expressed that victims seemed more able to manage or cope with their situation when the abuse was intermittent due to either party’s commitments or activities outside the home environment, but pandemic-related confinement meant that abuse, or the threat of abuse, was more constant. There were therefore significant mental health implications for callers, with a reported increase in calls where anxiety, depression and suicidal thoughts were a prominent theme. One participant in our study did acknowledge the impact of the pandemic on the abusive partner’s mental health, which potentially contributed further to the pattern of abuse during lockdown. Previous authors have also suggested this (e.g., Peterman et al., 2020), but more research into abuser experiences during the pandemic is required to identify the mechanisms that may have facilitated an increase in abusive behaviour.

As also highlighted in recent reports (e.g., Office for National Statistics, 2020), the mental health impact of living with abuse during the pandemic meant that helplines were required to directly engage in more safeguarding work than usual, often referring callers to mental health services such as the Samaritans or formalised counselling support. Some DA organisations began to offer in-house counselling support during the pandemic (Scottish Government, 2020), but demand was substantial and often unmatched to the physical or economic resources available to these charities. While the UK Government provided access to crisis funding for support organisations during this time, the level of funding was seen to be inadequate and many services remain concerned that the time-restricted nature of these grants mean that additional services will need to be withdrawn, discounting the longer-term pandemic impact on DA victims and expected continuous increase in service demand (Women’s Aid, 2020a). This highlights the importance of multi-agency working whereby resources and workload can be shared through appropriate referral pathways to provide holistic and comprehensive support to victims amidst and following termination of abusive relationships. For example, the Multi-Agency Risk Assessment Conferences (MARACS) mentioned earlier provide a crucial platform for information sharing between representatives from relevant agencies in any given jurisdiction. This will often include representatives of the local police force, healthcare, children and young people’s services, housing, education, probation, refuge, drug and alcohol services, and any other relevant statutory or voluntary organisation. Within these meetings, representatives work collaboratively to develop co-ordinated action plans to help reduce risk of harm to high-risk victims of domestic abuse. It is estimated that for every £1 spent on MARACs, at least £6 of public funds are saved on direct costs to agencies each year, demonstrating the impact that a multi-agency approach can have on the wider economic cost of DA (SafeLives, 2010). While MARACs are reserved for high-risk cases, through such multi-agency partnerships DA support organisations can build stronger relationships with other local agencies and create a clear map of appropriate referral pathways and processes to help ensure their service users can be safeguarded swiftly, without additional cost to the charities themselves.

### Limitations and Future Directions

While this study provides a timely and novel contribution to our understanding of the experiences of those living with DA during the COVID-19 pandemic from the perspective of DAH staff, it is not without its limitations. As this study was targeted at DA organisations within the UK, and given the relatively modest sample size, the application of findings to a wider non-UK context is somewhat limited. Furthermore, while DAH staff were at the forefront of support provision during the pandemic, call-takers often have time-limited interactions with service users. This limits the insight available from this group with regards to the more enduring, long-term impact of COVID-19 lockdown on those living with abusive partners. For this reason, it is imperative that the experiences of DA victims themselves are explored in more depth to provide further understanding of the abuse experienced, and long-term support needed going forward as we emerge from the pandemic to support this vulnerable group. Phase 2 and 3 of this study aims to do exactly this through anonymous online surveys and 1:1 interviews with victims of DA. Despite the limitations, this study provides a much-needed steppingstone to understanding how pandemic-related restrictions influenced the lives of those living with DA.

## Conclusions

While evidence indicates that COVID-19 and its associated restrictions placed substantial strain on intimate partners, and their relationships, across the general population (Goodboy et al., 2021; Pauley et al., 2022), this added strain undoubtedly had more severe consequences for those living with DA. While the circumstances throughout 2020–2021 severely limited the potential for safely acquiring first-person narratives from those living with DA, DAH staff were able to provide crucial third-party insight into the experiences of this vulnerable group in the interim. These insights highlighted staffs’ belief that the coronavirus pandemic and its associated restrictions presented a unique opportunity for abusers to intensify, prolong and justify their abusive behaviours. This was said to be exacerbated by restricted critical external abuse monitoring by friends and family, limited awareness or opportunities to safely access remote modes of support, and a lack of respite or relief from the abusive environment, leaving those living with DA at an exponential risk and further jeopardising their physical and mental wellbeing. Engagement with helpline support services did substantially increase during lockdown periods, indicating that many victims were aware of these services, were able to reach out safely, and found solace in their provision. However, issues have been highlighted in terms of caller clarity about services available and the accessibility of those services for the diverse population of DA victims. Furthermore, there seemed to be limited awareness of exceptions to government restrictions for those living with abuse, leaving callers feeling unable to leave the home at any time, or contact friends and family. This emphasizes how important inclusivity and widespread public awareness of support mechanisms and services are moving forward.

While DA support services made a monumental effort to provide support for those locked down with abusive partners, DA support services in the UK were already argued to be inadequate due to funding limitations prior to COVID-19 (Bradley, 2020). This indicates that, while this problem may have been aggravated by lockdown, it is not localised to the pandemic and will continue to require urgent attention from support organisations and policy decision makers post-COVID. Particularly, the reported mental health impact on those living with an abusive partner during lockdown will be long-lasting, and so funding must be made available to support services to allow for the continued response to the pandemic-related surge in DA. However, given that relying on consistent financial support from government is often not viable, focus needs to be driven towards campaigns that can have legacy impact whether or not the same level of funding is available. For example, efforts to increase public awareness of the many forms that domestic abuse can take, what support avenues are available and how they can be pursued (e.g., refuge) and what the long-term impact of DA could be can help contribute to an improved community-wide response to DA. Given the growing pressure faced by DA support organisations, it is also crucial that they continue to build and maintain multi-agency partnerships, especially with mental health services, and that staff are provided with extensive training to ensure awareness of appropriate referral pathways and procedures to safeguard vulnerable service users.

Finally, this research revealed some benefits to the remote services provided by organisations during this time, including increased accessibility for those who would have ordinarily struggled to make it on-site, and more diversity in the remote contact options available (e.g., expansion of webchats, email, and text support). For organisations who were not offering these services before, the pandemic prompted more in-depth consideration of the limited opportunities that a victim might have to make a lengthy phone call. While stay-at-home orders have now been withdrawn, there are many who will continue to live much of their day in close proximity to their abuser, emphasising the ongoing importance of support mediums that minimise the risk of detection. It is therefore heavily advised that, in addition to the reintroduction of face-to-face support options, the increased provision of diverse virtual support options is maintained post-pandemic, especially such mediums that allow service users to easily leave and return to conversations when they are able (e.g., webchats, text support). However, caution must be taken in deliberations as to the longevity of the exclusively virtual support approach devised as a response to COVID-19 restrictions as we continue to emerge from the pandemic. The findings of this paper with regards to the negative implications of purely virtual support for this vulnerable group should be taken into consideration in risk assessment and decision-making should we face further lockdowns, with the recommendation that DA support organisations should be considered an exception to any in-person contact restrictions.
